# Evaluation and comparison of New 4DCT based strategies for proton treatment planning for lung tumors

**DOI:** 10.1186/1748-717X-8-73

**Published:** 2013-03-25

**Authors:** Ning Wang, Baldev Patyal, Abiel Ghebremedhin, David Bush

**Affiliations:** 1 Department of Radiation Medicine, Loma Linda University Medical Center, Loma Linda, CA 92354, USA

**Keywords:** Proton radiotherapy, Proton lung treatment planning, 4DCT scan

## Abstract

**Purpose:**

To evaluate different strategies for proton lung treatment planning based on four-dimensional CT (4DCT) scans.

**Methods and Materials:**

Twelve cases, involving only gross tumor volumes (GTV), were evaluated. Single image sets of (1) maximum intensity projection (MIP3) of end inhale (EI), middle exhale (ME) and end exhale (EE) images; (2) average intensity projection (AVG) of all phase images; and (3) EE images from 4DCT scans were selected as primary images for proton treatment planning. Internal target volumes (ITVs) outlined by a clinician were imported into MIP3, AVG, and EE images as planning targets. Initially, treatment uncertainties were not included in planning. Each plan was imported into phase images of 4DCT scans. Relative volumes of GTVs covered by 95% of prescribed dose and mean ipsilateral lung dose of a phase image obtained by averaging the dose in inspiration and expiration phases were used to evaluate the quality of a plan for a particular case. For comparing different planning strategies, the mean of the averaged relative volumes of GTVs covered by 95% of prescribed dose and its standard deviation for each planning strategy for all cases were used. Then, treatment uncertainties were included in planning. Each plan was recalculated in phase images of 4DCT scans. Same strategies were used for plan evaluation except dose-volume histograms of the planning target volumes (PTVs) instead of GTVs were used and the mean and standard deviation of the relative volumes of PTVs covered by 95% of prescribed dose and the ipsilateral lung dose were used to compare different planning strategies.

**Results:**

MIP3 plans without treatment uncertainties yielded 96.7% of the mean relative GTV covered by 95% of prescribed dose (standard deviations of 5.7% for all cases). With treatment uncertainties, MIP3 plans yielded 99.5% of mean relative PTV covered by 95% of prescribed dose (standard deviations of 0.7%). Inclusion of treatment uncertainties improved PTV dose coverage but also increased the ipsilateral mean lung dose in general, and reduced the variations of the PTV dose coverage among different cases. Plans based on conventional axial CT scan (CVCT) gave the poorest PTV dose coverage (about 96% of mean relative PTV covered by 95% isodose) compared to MIP3 and EE plans, which resulted in 100% of PTV covered by 95% isodose for tumors with relatively large motion. AVG plans demonstrated PTV dose coverage of 89.8% and 94.4% for cases with small tumors. MIP3 plans demonstrated superior tumor coverage and were least sensitive to tumor size and tumor location.

**Conclusion:**

MIP3 plans based on 4DCT scans were the best planning strategy for proton lung treatment planning.

## Introduction

Proton treatment for lung cancer can spare or minimize dose to contralateral lung tissue. We have been treating medically inoperable non-small-cell lung carcinoma (NSCLC) for more than ten years [1-3]. We have used three regimens: (a) X-rays followed by proton boost, with a conventional fraction rate of 1.8 Gy per day; (b) protons only, with a fraction rate of 1.8/2.0 Gy per day; (c) hypofractionated protons, with a fraction rate of 6/7 Gy per day in 10 fractions. Patients were assigned to one of the protocols based on their medical condition and tumor grade. Bush et al. [1] followed up 37 patients with early-stage NSCLC and reported better survival and local control using proton therapy with an updated review published in 2010 [2]. Moyers et al. [4] studied the methodologies and tools for proton lung treatment planning, and pointed out that a proton beam design should include tumor motion and setup uncertainties instead of creating new targets. That study introduced aperture and compensator design using margins and smearing to take into account tumor motion and setup uncertainties. The smearing method was similar to the compensator design proposed by Urie et al. [5] to account for setup uncertainties and tumor motion.

We have used conventional axial CT (CVCT) images for proton lung treatment planning before four-dimensional CT (4DCT) became available. Severe motion artifacts can be introduced during acquisition of CVCT images for a moving target [6-8]. Such artifacts distort the volume of structures when substantial motion occurs. The artifacts, in turn, adversely affect proton lung treatment planning. 4DCT synchronizes the time-resolved CT data acquisition with respiratory motion monitored by a real-time position management (RPM) system. In the cine mode of 4DCT scanning, the CT table keeps the same position in a breathing cycle of a patient. Multiple images are reconstructed for each table position. Images of the same breathing phase at different CT table positions are sorted together into different phase images. A breathing cycle can be divided into different phases (0%, 30%, 50%, 70% and 90%), which represent the end-inhale (EI), middle-exhale (ME), end-exhale (EE), middle-inhale (MI), and near-end-inhale (NEI) phases of a breathing cycle. Each phase corresponds to a near static moment in the breathing cycle of a patient. The corresponding images of each phase contain fewer motion artifacts.

Engelsman et al. [9] have proposed a proton lung treatment planning strategy based on 4DCT scanning. It combines three plans using EI, ME and EE images into a single treatment plan. This technique requires special tools in the treatment planning system to combine multiple plans from different phase images. Although it gives acceptable results, it is not clinically efficient. Kang et al. [10] compared four planning strategies based on images of: (1) AVG, (2) free breathing helical CT, (3) MIP, and (4) AVG with the density correction of a moving tumor (AVG_RIGTV). They suggested that the AVG_RIGTV approach be employed for proton lung treatment planning. This approach requires a uniform density substitution of 100 HU (water = 0 HU) for the entire IGTV. This approach ensures coverage of the tumor but delivers a higher dose to the surrounding tissue. Moreover, uniform density substitution will have different effect on treatment planning outcome for different tumor sizes and locations.

Our clinical outcomes and experiences of proton lung treatment were based on CVCT plans. Herein we compare proton lung treatment planning using 4DCT scans against plans derived from this benchmark. MIP3, AVG and EE images from 4DCT scans were selected as the primary images for planning. ITVs outlined by a clinician were exported from the original treatment plans and imported into MIP3, AVG, and EE images as the planning targets. Initially there were no uncertainties included in the planning process. This was done to compare different planning strategies without institution specific preferences for treatment planning uncertainties. Later, CT number, treatment setup, and proton beam range uncertainties were included in the plans. The plans were imported and recalculated in phase images of 4DCT scans for plan evaluation and comparison of different planning strategies. We will show that MIP3 planning strategy, introduced here, gives the best overall tumor coverage, and surrounding tissue sparing, irrespective of the magnitude of tumor motion, tumor size or location.

## Material and methods

### CVCT plan

The treatment planning system employed at our institution (Odyssey 4.8; Optivus Proton Therapy), automatically selects proton beam energy and modulation, calculates proton beam range shift, and generates an aperture and compensator for each portal to cover a target laterally and distally. The aperture is shaped by the target with a margin for proton beam penumbra and treatment setup uncertainty. The compensator is designed by the straight-line ray tracings of water-equivalent depths in the target. The thickness of a compensator at each ray line is the difference between the radiological path length of the 90% distal dose fall-off of a proton beam and the water equivalent depth of a target at the distal end. The smearing in the compensator design compares the thickness of the compensator at a ray line with its surrounding ray lines in a radius and chooses the smallest value as the thickness of the compensator at the ray line. This radius, called the smearing radius, is determined by tumor motion, proton beam range, and treatment setup uncertainty. The smeared compensator increases the penetration of the proton beam and ensures distal coverage of the tumor in the presence of tumor motion and treatment setup uncertainties.

**Figure 1:**
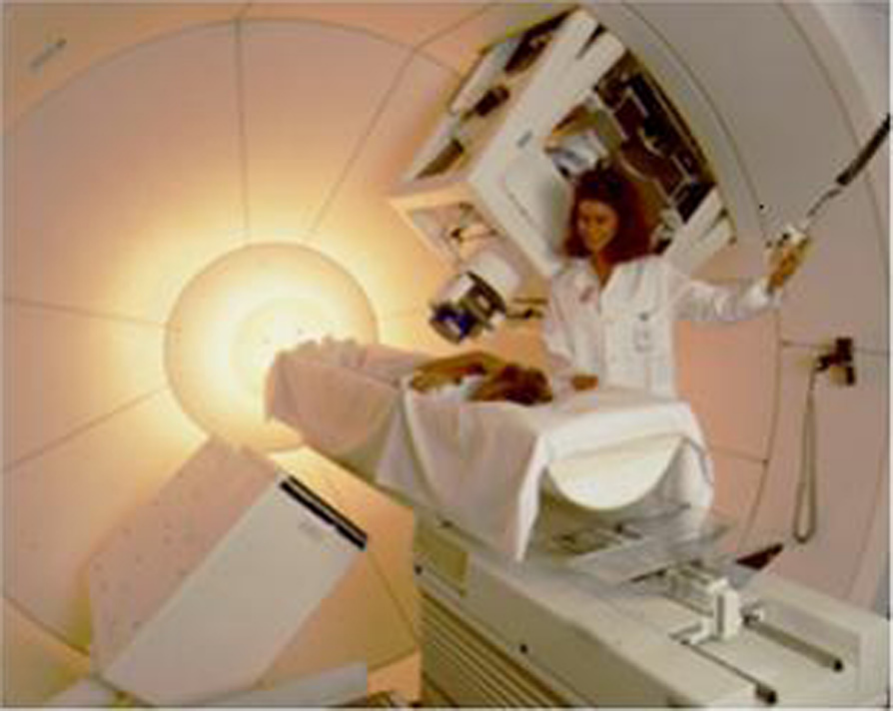
**A typical proton treatment set up.** Patient is in the whole body pod. Blue wax compensator and digital image receptor are visible in the figure.

A patient is immobilized supine in a whole-body pod for CT scanning and subsequent proton lung treatment (Figure 1). The 4DCT scan is performed by a GE 64-slice LightSpeed QX/I CT scanner in the cine mode. A Varian RPM system records the respiratory motion. The 4DCT scan and respiratory profile are temporally correlated. EI, ME, EE, MI, and NEI images are retrospectively sorted according to respiratory phases. CVCT images are acquired by conventional axial CT scan in free-breathing mode. Twelve patients, who received proton lung treatments for NSCLC at our institution with different tumor sizes and locations, were selected for the study. The cases, named A-L, were treated with either 60 Gy in 30 fractions or 60/70 Gy in 10 fractions. Proton lung treatments were planned using CVCT images. MIP images as alternate studies were correlated with CVCT images to outline ITVs as planning targets by a clinician. Treatment setup, proton beam range, and CT number uncertainties were included in the plans. Apertures were shaped by ITVs with margins for proton beam penumbra and treatment setup uncertainty. Compensators were designed to stop proton beams at the distal edges of ITVs, allowing margins for distal dose fall-off of proton beams. The smearing used in the compensator design was determined by the maximum motion of ITVs, treatment setup uncertainty, and 3% of proton beam range for proton scattering [4]. The smearing compensated for the density variations of ITVs and motion artifacts in CVCT images that contained the randomized superimposed moving targets.

All cases contained only one target, except case J, which had two lesions nested in the upper and middle lobes of the left lung. Table 1 indicates the locations of all the tumors, their motion characteristics, and the tumor size in the EE phase. The tumor of case L was nested along the anterior side of the lung and adjacent to the chest wall and heart. In the remaining cases, tumors were located along the posterior or middle side of lung. In terms of breathing patterns, L and G represented extreme cases with breathing periods of 1.2 and 9.0 seconds, respectively. The remaining cases had breathing periods of about 3 to 5 seconds. For most cases expiration was longer or equal to inspiration; cases A, D, and L being notable exceptions. Patients generally stay near EE phase longer. Tumor volumes in the EE phase varied from 0.4 to 95.4 cubic centimeters (cc), as listed in Table 1. Tumor motion in three dimensions (3D) was calculated by r.m.s of motions in the left-right, superior-inferior and anterior-posterior directions. Cases A, B, and E had similar tumor motions in 3D, i.e., of about 3 mm; these motions were less than the rest of cases except case L, which had the largest tumor volume and minimum tumor motion in 3D. Case F had the maximum tumor motion of 31 mm along the superior-inferior direction. Case K had the largest tumor motion in the anterior-posterior direction of 7.4 mm. Comparing tumor volumes in EE images, cases A through C had small tumor volumes of less than 2 cc; cases D through G had tumor volumes of 7 to 8 cc; cases H through J had tumor volumes of 18 to 26 cc; and cases K and L had tumor volumes of 70 and 95 cc.

**Table 1:** Tumor location, inspiration and expiration durations, tumor volumes in EE phase, tumor motion between EI and EE phases, and in 3D

**Cases**	**Tumor location**	**Inspiration (seconds)**	**Expiration (seconds)**	**Tumor volume in EE phase (cc)**	**Motion (mm) between EI and EE phases**			**Tumor motion in 3D (mm)**
					**Left-right**	**Superior-inferior**	**Anterior-posterior**	
A	Right upper lobe	1.6	0.5	0.4	1.5	3.2	1.3	2.2
B	Right upper lobe	1.7	2.4	1.3	3	3	2	2.7
C	Left upper lobe	1.5	1.5	2	1.1	6.3	4.0	4.4
D	Right upper/middle lobe	2	1.8	6.5	1.5	8	5.5	5.7
E	Right upper lobe	1.2	3.3	7.4	1.6	3.4	1.2	2.3
F	Right middle lobe	1.5	2.5	7.7	7.2	31.0	8.6	19.0
G	Left upper lobe	3.7	5.3	8.1	2.0	13	12	10.3
H	Left lower lobe	1.4	1.5	17.9	1.3	24.4	5.5	14.5
I	Left lower lobe	1.5	2.4	20.0	0.6	15.7	0.5	9.1
J	Left upper/lower lobe	2.3	2.3	25.7	2.1	10.7	6.3	7.3
K	Right upper lobe	1.7	2.1	69.8	4.3	5.1	7.4	5.8
L	Left upper/lower lobe	0.7	0.5	95.4	1.6	2.3	1.1	1.7

### Primary Images and ITVs for proton treatment planning

**Figure 2:**
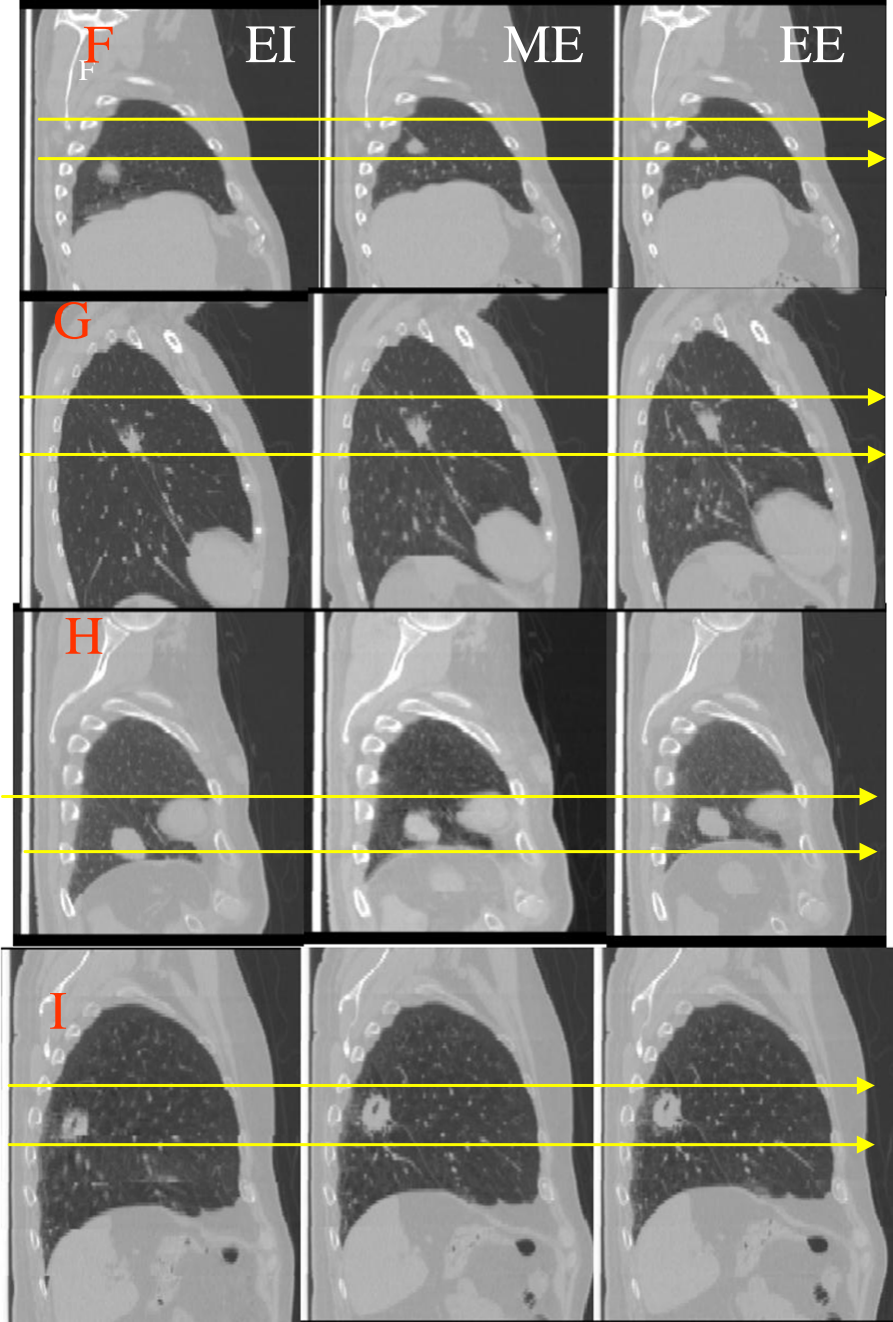
**Tumor positions and lung volumes in EI, ME and EE images for cases F, G, H and I.** The double lines correspond to the tumor positions in ME phase.

**Figure 3:**
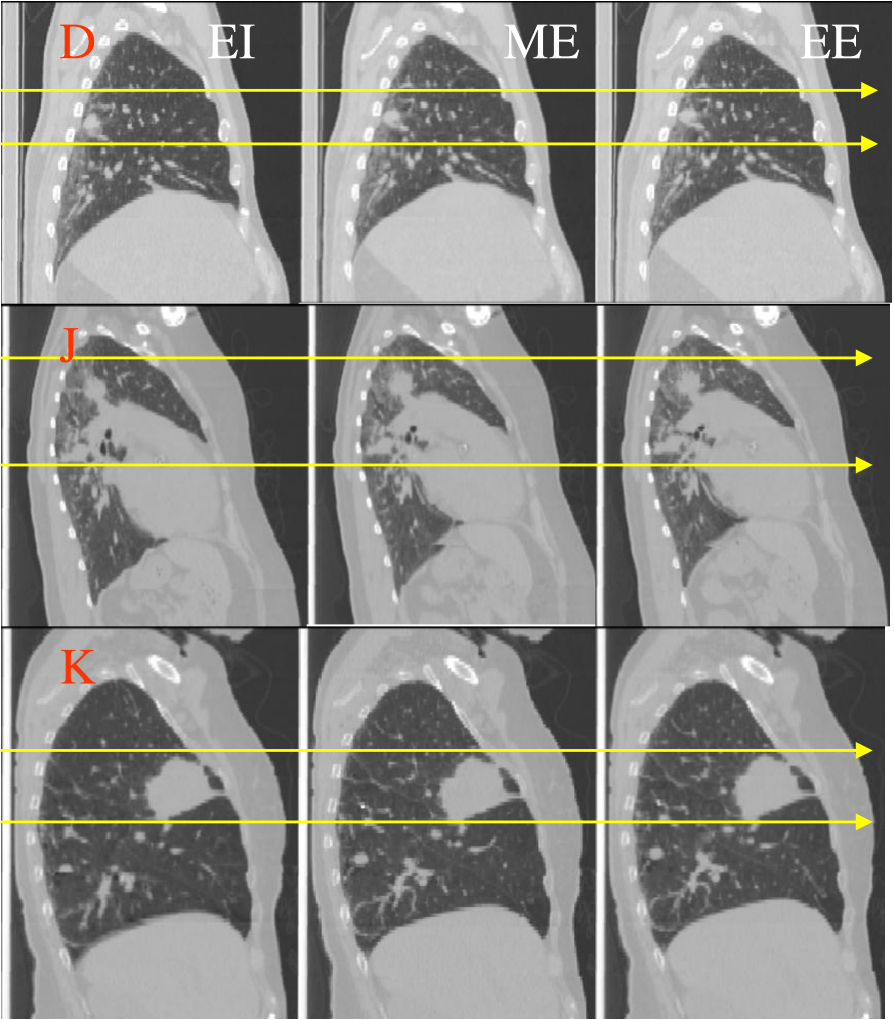
Tumor positions and lung volumes in EI, ME and EE images of case D, J and K, the double lines correspond to the tumor positions in ME images.

MIP images have artificially higher tissue densities and thus were not selected as primary images for proton planning. Instead, the MIP images from EI, ME, and EE phases, named as MIP3 images, and introduced in this study, were used as primary images for treatment planning. MIP3 and MIP images are based on the same principle of image reconstruction, but MIP3 images contain the magnitude of tumor motion but do not artificially increase the tissue densities as seen in MIP images. Lung tissue generally contains its maximum air volume in the EI phase and its minimum air volume in the EE phase. Tumors, thus, might show greater movement from the EI to ME phase than from the ME to EE phase because of the faster outward movement of air volume immediately after the EI phase. Figure 2 displays the comparison of lung volumes for different tumor positions relative to their anatomy in EI, ME, and EE phases for case F, G, H, and I. There were relatively larger tumor motions from the EI to ME than from the ME to EE phases, which indicated that tumors with large motion stayed near the EE phase longer. For 3D tumor motion of less than 8 mm, as in cases D, J, and K, shown in Figure 3, there was relatively less tumor movement from the EI to EE phase. Accordingly, EE images were chosen as primary images from 4DCT scanning for proton lung treatment planning comparisons. Previous studies [10] demonstrated that AVG images with density correction of moving targets were better for proton lung treatment planning. We selected (1) MIP3, (2) AVG, and (3) EE images as the primary images from 4DCT scans for this planning study. ITVs from CVCT plans were imported into MIP3, AVG, and EE images as planning targets. The corresponding plans for MIP3, AVG, and EE were compared with CVCT plans for quality evaluation of a planning strategy.

### Treatment planning without uncertainties

The cases were planned with posterior-anterior and left-right lateral beams, except case H, which used posterior-anterior and left-anterior-oblique beams. Treatment setup, CT number, and proton beam range uncertainties were excluded from the plans in order to compare different planning strategies independent of all institution specific uncertainties. The aperture of each portal was shaped by ITVs with a margin equal to the proton beam’s 95% to 50% penumbra for lateral dose fall-off. The compensator of each portal was designed to have 95% distal dose coverage of the ITVs. ITVs from EE images had the outline of moving targets and contained tumor densities in the EE phase only. ITVs from AVG images contained superimposed moving tumors with averaged tissue densities lower than solid tumors in the EE phase. ITVs from EE and AVG images required density corrections for the compensator design in the EE and AVG plans. The density of a solid tumor in the EE phase was used to replace densities of ITVs in EE and AVG images to ascertain adequate beam penetration. ITVs from MIP3 images were the superimposed tumors from EI, ME, and EE phases, which contained tumors at the middle and two extreme phases of a breathing cycle. The density variations of tumors in between other phases had to be compensated for ITVs as planning targets in the MIP3 plans. The smearing technique was applied in the compensator design of MIP3 plans. Smearing radii for MIP3 and CVCT plans were calculated as follows:

SRMIP3=0.03×Range2+12MaxMotionEI,ME2

SRCVCT=0.03×Range2+12MaxMotionCVCT,MIP2

Where *SR*_*MIP3*_ and *SR*_*CVCT*_ were the smearing radii used for MIP3 and CVCT plans, *MaxMotion*_*EI, ME*_ and *MaxMotion*_*CVCT, MIP*_ were the maximum tumor motion perpendicular to a beam direction between EI and ME, and CVCT and MIP images, and *Range* represented the proton beam range. In the equations above, 3% of proton beam range accounted for proton beam scattering along its path; the second part compensated for tumor motion in the compensator design of MIP3 and CVCT plans. The smearing radius of MIP3 plans was calculated from the maximum tumor motion in between the EI and ME phase. This was done because lung tumors generally have greater movement from EI to ME phases than from ME to EE phases**.** The smearing radius between the EI to ME phase will not only compensate for tumor movements between the EI to ME phase, but also between the ME to EE phase.

CVCT images contain randomly superimposed moving tumors during image acquisition by axial CT scan. The smearing radius of CVCT plans used the maximum tumor motion between CVCT and MIP images to compensate for tumor movements not captured by CVCT images. The smearing radius of a MIP3 plan was dominated mainly by the large tumor motion along the superior-inferior direction between the EI and ME phases. The smearing radii of CVCT plans of cases F through J were larger than those of MIP3 plans. The maximum tumor motions between CVCT and MIP images were larger compared to tumor motion between the EI and ME phases of cases F through J. The smearing radii of CVCT and MIP3 plans of cases A, B, C, and E were smaller than those of cases F through J. The small tumor motion of cases A, B, C, and E decreased the differences of the smearing radii between CVCT and MIP3 plans. The smearing radii of CVCT and MIP3 plans of cases K and L were mainly dominated by proton beam scattering along the beam paths.

For each case, CVCT, MIP3, AVG, and EE plans were fixed and imported into EI, ME, EE, MI, and NEI images for the plan evaluation, in which GTVs and the ipsilateral lung were outlined. The ipsilateral lung excluded GTVs. The mean dose and dose-volume histograms of lung were calculated. GTV dose instead of PTV dose was used for plan evaluation because no treatment uncertainties were included at this stage in the planning process. The relative volumes of GTVs covered by 95% of prescribed dose and mean lung dose from EI, ME, EE, MI, and NEI images were averaged by the inspiration and expiration duration of a case in a breathing cycle as follows:

$$\begin{array}{l} GV_{95}=\{\mathrm{InD}\times \left(V_{95}\mathrm{EI}+V_{95}\mathrm{MI}+V_{95}\mathrm{NEI}\right)/3+\mathrm{ExD}\times \\ \left(V_{95}\mathrm{EE}+V_{95}\mathrm{ME}\right)/2\}/\left(\mathrm{InD}+\mathrm{ExD}\right) \end{array}$$

$$\begin{array}{l} GM_{\mathrm{LD}}=\{\mathrm{InD}\times \left(M_{\mathrm{LD}}\mathrm{EI}+M_{\mathrm{LD}}\mathrm{MI}+M_{\mathrm{LD}}\mathrm{NEI}\right)/3+\mathrm{ExD}\times \\ \left(M_{\mathrm{LD}}\mathrm{EE}+M_{\mathrm{LD}}\mathrm{ME}\right)/2\}/\left(\mathrm{InD}+\mathrm{ExD}\right) \end{array}$$

Where *GV*_*95*_ and *GM*_*LD*_ were, respectively, the averaged relative volumes of GTVs covered by 95% of prescribed dose and mean lung dose from EI, ME, EE, MI, and NEI images; *InD* and *ExD* were the inspiration and expiration durations; *V*_*95*_*EI*, *V*_*95*_*MI*, *V*_*95*_*NEI*, *V*_*95*_*EE,* and *V*_*95*_*ME* represented the relative volume of GTVs covered by 95% of prescribed dose in the EI, MI, NEI, EE, and ME phases; and *M*_*LD*_*EI*, *M*_*LD*_*MI*, *M*_*LD*_*NEI*, *M*_*LD*_*EE*, and *M*_*LD*_*ME* were the mean lung doses in the EI, MI, NEI, EE, and ME phases. *GV*_*95*_ and *GM*_*LD*_ displayed the average tumor volume covered by 95% of the prescribed dose and mean lung dose in a breathing cycle. In addition, we calculated the mean averaged relative volumes of GTVs covered by 95% of prescribed dose for all cases in the study (*MGV*_*95*_) to display the overall tumor dose coverage for a planning strategy. The standard deviation (*STDGV*_*95*_) of a planning strategy from *MGV*_*95*_ was calculated as well.

### Treatment planning including uncertainties

In this case, treatment planning uncertainties, viz., treatment setup, CT number, and proton beam range uncertainties were included in CVCT, MIP3, AVG, and EE plans. The treatment setup uncertainty for proton lung treatment using a whole-body pod is about 5 mm, which increased the lateral margins between aperture and ITVs, and increased the smearing of compensators. A smearing radius of 5 mm was used in the compensator design of AVG and EE plans for compensating the setup uncertainty in the plans. To account for the setup uncertainty, the smearing radii for CVCT and MIP3 plans were increased by 5 mm in addition to the already calculated *SR*_***CVCT***_ and *SR*_***MIP3.***_ The distal margin of ITV for the compensator design was calculated as

0.035 × *ITV distal depth*(*water equivalent*) + 3 *mm*, where first part represents CT number uncertainty and the second part represents proton beam range uncertainty. The plans were imported into and recalculated in EI, MI, NEI, EE, and ME phase images, in which PTVs were derived from GTVs with the margin equal to treatment setup uncertainty in the direction perpendicular to proton beam direction. From each of CVCT, MIP3, AVG, and EE plans, dose volume histograms of PTVs and those of ipsilateral lung in EI, ME, EE, MI, and NEI phases were calculated by averaging over the inspiration and expiration duration in a breathing cycle as discussed above. The averaged relative volumes of PTVs covered by 95% of prescribed dose (*PV*_*95*_), mean lung dose (*PM*_*LD*_), and relative volumes of ipsilateral lung receiving more than 20 Gy (*LV*_*20*_) from CVCT, MIP3, AVG, and EE plans were compared. The mean *PV*_*95*_ of all the cases (*MPV*_*95*_) and standard deviation (*STDPV*_*95*_) of *PV*_*95*_ were calculated for each CVCT, MIP3, AVG, and EE planning strategy for all cases. These calculations were carried out to demonstrate the overall PTV dose coverage and sensitivity of a particular planning strategy to different tumor sizes and locations in the presence of treatment uncertainties which are included in the planning process. The optimal planning strategy was selected based on this analysis.

To further evaluate the robustness of our technique we planned two cases, case A, smallest tumor, and case F, largest motion, under over-irradiate and under-irradiate modes. Under the over-irradiate mode the beam is allowed to penetrate deeper by 0.035 x ITV depth (water equivalent depth) + 3 mm, and for the under-irradiate mode the beam is retracted by the same amount. It is in addition to the uncertainties already included in planning for the PTV. This was done to create extreme situations and see how MIP3 compares with other strategies under these conditions.

## Results

Table 2 lists the average relative volumes of GTVs receiving 95% of prescribed dose, and the average mean ipsilateral lung dose in EI, MI, NEI, EE, and ME phases from CVCT, MIP3, AVG, and EE plans in the absence of uncertainties. Cases A and B had less than 95% of the average relative volumes covered by 95% of prescription dose for all plans. MIP3 planning showed the best overall coverage by the 95% isodose covering more than 95% of GTV for all cases from C to L, resulting in the best *MGV*_*95*_ of 96.7% and lowest standard deviation (5.7%). AVG plans performed worst, with the lowest *MGV*_*95*_ of 93.6% and highest standard deviation (10.4%). These results suggested that the MIP3 plan yielded superior tumor coverage irrespective of tumor size and location. On the other hand, all planning strategies delivered similar lung dose (*GM*_*LD*_) for every case studied; but dose varied with tumor size and location. Irradiation of large tumors (case K and L, Table 1) resulted in higher dose to normal lung (Table 2).

**Table 2:** **Averaged relative volumes of GTVs covered by 95% of prescription dose (*GV***_***95***_**) for different planning strategies**

	**Case**	**A**	**B**	**C**	**D**	**E**	**F**	**G**	**H**	**I**	**J**	**K**	**L**	***MGV***_***95***_	***STDGV***_***95***_
*GV*_*95*_ (%)	CVCT plan	64.9	84.1	97.5	99.8	97.3	95.1	93.8	97.0	99.9	99.8	100	98.4	94.1	10.2
	MIP3 plan	82.2	88.3	97.1	100	96.9	100	96.3	100	100	99.8	99.7	100	96.7	5.7
	AVG plan	64.0	86.3	90.9	96.3	95.3	99.8	91.5	100	100	99.7	100	100	93.6	10.4
	EE plan	69.1	89.7	96.3	99.8	96.5	99.9	94.4	100	100	99.8	100	99.8	95.4	8.9
*GM*_*LD*_ (Gy)	CVCT plan	1.0	1.9	3.6	2.9	3.6	4.9	6.6	4.1	5.6	7.3	11.1	11.7		
	MIP3 plan	1.1	2.0	3.7	3.0	3.8	5.1	6.9	4.5	5.3	7.3	11.5	11.0		
	AVG plan	0.9	2.0	2.9	2.5	3.4	4.1	5.5	4.0	4.5	6.2	11.3	11.0		
	EE plan	0.8	2.0	3.1	2.5	3.4	4.2	5.7	3.9	4.7	6.5	11.5	11.0		

Mean ipsilateral lung dose (*GM*_*LD*_) (Gy) from CVCT, MIP3, AVG and EE plans (treatment uncertainties not included).

**Figure 4:**
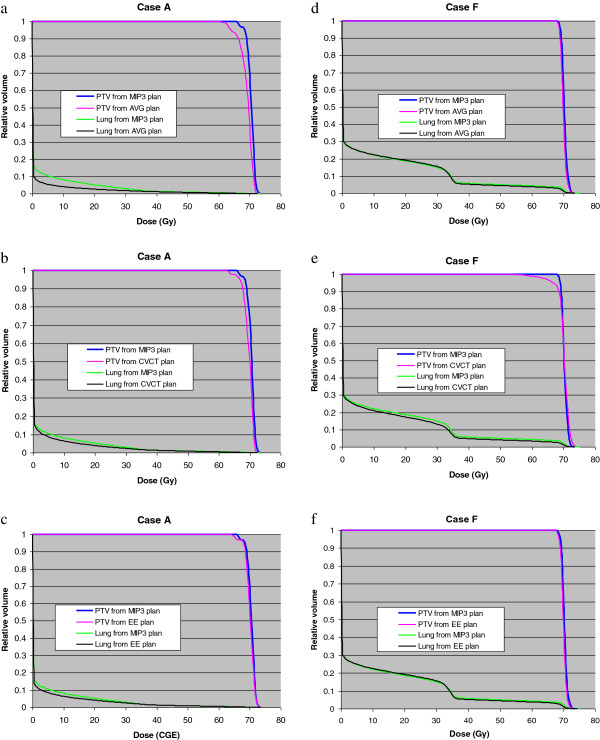
Comparison of MIP3 planning strategy with other planning strategies for the case of a tumor with smallest size (case A): (a) – (c); and for the case of a tumor with maximum motion (case F): (d) – (f).

Table 3 lists the average relative volumes of PTVs covered by 95% of prescribed dose (*PV*_*95*_) from CVCT, MIP3, AVG, and EE plans, with treatment uncertainties included in the planning process. The mean of the average relative volumes of PTVs covered by 95% of prescribed dose (*MPV*_*95*_) from each of the planning strategies and standard deviation (*STDPV*_*95*_) are also shown in Table 3. Results indicate that, in the presence of treatment uncertainties, MIP3 planning methodology gave the best PTV coverage, with better than 98% PTVs covered by 95% isodose line; case L being at the lower end at 97.8% coverage. Based on *MPV*_*95*_ and *STDPV*_*95*_ criteria, MIP3 planning yielded superior overall results as well. Although for certain cases other methodologies also performed satisfactorily, MIP3 planning demonstrated the least sensitivity to tumor location and tumor size, resulting in optimal tumor coverage as evidenced by the maximum *PV*_*95*_, *MPV*_*95*_ and least *STDPV*_*95*_. Also listed in Table 3 are the ipsilateral lung volumes receiving more than 20 Gy (*LV*_*20*_), and the mean lung dose (*PM*_*LD*_) from CVCT, MIP3, AVG, and EE plans averaged over the inspiration and expiration duration of each case. Based solely on *LV*_*20*_ and *PM*_*LD*_ criteria, no clear patterns emerged which could point toward a superior planning strategy. But combined with superior tumor coverage and least sensitivity to tumor size and location; MIP3 proved to be a winning strategy. For example, based on *LV*_*20*_ and *PM*_*LD*_ criteria; AVG plan gives best results for case A; but *PV*_*95*_ of only 89.8% makes this an unacceptable planning strategy (Table 3). As an illustrative example, Figure 4 shows the averaged dose volume histograms of PTVs and ipsilateral lung comparing MIP3 with other planning strategies for case A (smallest tumor) and case F (largest tumor motion).

**Table 3:** **Average relative volumes of PTVs covered by 95% of prescription dose (*PV***_***95***_**), and *MPV***_***95 ***_**and *STDPV***_***95***_**, and average ipsilateral lung volumes receiving more than 20 Gy (*LV***_***20***_**) and Mean lung dose (*PM***_***LD***_**) (Gy) from CVCT, MIP3, AVG and EE plans (treatment uncertainties included)**

	**Case**	**A**	**B**	**C**	**D**	**E**	**F**	**G**	**H**	**I**	**J**	**K**	**L**	***MPV***_***95***_	***STDPV***_***95***_
*PV*_*95*_ (%)	CVCT plan	96.1	98.5	100	99.9	99.5	95.4	95.6	99.8	99.9	99.7	98.9	97.8	98.4	1.7
	MIP3 plan	98.4	99.1	100	100	99.9	100	99.7	99.8	99.8	99.8	99.3	97.8	99.5	0.7
	AVG plan	89.8	94.4	100	100	100	100	98.5	99.8	99.8	99.8	99.5	97.0	98.2	3.1
	EE plan	96.8	99.7	100	99.9	100	100	99.6	99.9	100	99.8	99.8	97.4	99.4	1.1
*LV*_*20*_ (%)	CVCT plan	3.9	8.0	13.6	7.6	13.7	18.7	23.8	12.1	20.0	25.7	32.3	30.3		
	MIP3 plan	5.1	8.4	15.1	9.3	13.7	18.6	23.0	11.5	18.2	25.9	32.8	30.8		
	AVG plan	2.6	7.1	13.4	7.9	13.2	19.0	20.5	11.9	19.9	25.7	33.4	31.0		
	EE plan	4.0	8.2	13.4	7.9	13.2	19.1	21.3	11.2	21.0	25.8	33.1	31.0		
*PM*_*LD*_ (Gy)	CVCT plan	2.4	3.9	6.7	4.3	6.3	8.4	10.8	6.7	9.1	13.0	15.7	14.7		
	MIP3 plan	2.8	4.0	7.1	5.1	6.2	8.8	10.1	6.5	8.4	12.7	16.0	15.0		
	AVG plan	1.7	3.4	6.6	4.4	6.1	8.6	9.6	6.5	8.8	12.7	16.3	15.1		
	EE plan	2.4	3.9	6.5	4.4	6.1	8.7	9.9	6.2	9.2	12.8	16.5	15.2		

Table 4 lists *PV*_*95*_ and *PM*_*LD*_ for cases A (smallest tumor) and F (largest motion) using over-irradiate and under-irradiate modes for different planning strategies. For comparison the results for nominal irradiation (as described earlier) are also listed. As can be seen from the results in Table 4, even under these extreme situations (highly unlikely but not improbable), MIP3 planning strategy fares as well as or better than other strategies. Also, as can be seen from the results in Table 4, under-shooting is more serious a problem in proton therapy than over-shooting. Since a proton beam stops abruptly near the end-of-range, the portion of the tumor beyond the distal edge of the Bragg peak, as happens in under-shooting, is left virtually cold, leading to serious under-dosing, especially, for small tumors.

**Table 4:** **Average relative volumes of PTVs covered by 95% of prescription dose (*PV***_***95***_**), and Mean lung dose (*PM***_***LD***_**) (Gy) from CVCT, MIP3, AVG and EE plans under conditions of nominal, over irradiation and under irradiation**

	**Case**	**A**			**F**		
	**Irradiate mode**	**Over**	**Under**	**Nominal**	**Over**	**Under**	**Nominal**
*PV*_*95*_ (%)	CVCT plan	95.6	77.4	96.1	100	91.0	95.4
	MIP3 plan	96.9	96.0	98.4	100	98.4	100
	AVG plan	95.7	73.7	89.8	100	99.7	100
	EE plan	95.7	76.3	96.8	100	99.9	100
*PM*_*LD*_ (Gy)	CVCT plan	2.9	1.6	2.4	8.7	6.3	8.4
	MIP3 plan	3.0	1.7	2.8	9.0	7.0	8.8
	AVG plan	2.9	1.6	1.7	9.0	7.3	8.6
	EE plan	2.9	1.7	2.4	9.1	7.4	8.7

## Discussion

Our study compared different treatment planning strategies using 4DCT data to accommodate motion in proton lung treatment planning. Plans were done with and without treatment uncertainties. As shown in Table 2, when uncertainties are not included all plans demonstrated similar GTV dose coverage for cases H through L. These cases represent relatively large tumor volumes. On the other hand, cases involving small tumor sizes (A through E) showed large variations in GTV dose coverage. The challenge for a good planning strategy comes from its capability of providing adequate dose coverage to small tumors in the presence of motion. Cases A and B, with the smallest tumor volumes (Table 1), showed the worst GTV dose coverage regardless of planning strategy (Table 2) because small tumors are more sensitive to movements and deformation. AVG plans yielded inferior GTV dose coverage for cases C through E compared to MIP3, EE, and CVCT plans. This result, and the results of cases A and B, indicate that an AVG plan strategy needs higher density corrections of the ITVs for small tumors to compensate lower tissue densities along proton beam paths of AVG images. As can be seen in Table 2, MIP3 plans, overall, resulted in the best GTV dose coverage (GV_95_) and the highest mean GTV dose (MGV_95_) with the minimum standard deviation (STDGV_95_) for all cases studied.

Inclusion of treatment uncertainties increased the lateral and distal margins of ITVs. The increased distal margins of ITVs increased the proton beam penetration via increased smearing in the compensator design. The increased margins improved the PTV dose coverage, but they also increased dose to the ipsilateral lung compared to the plans without treatment uncertainties (Tables 3). As shown in Table 3, the PTV dose coverage of cases C through E was improved compared to the GTV dose coverage from the plans without treatment uncertainties. For large tumors (cases H through L), dose coverage with and without uncertainties was very similar (within 2%). On the other hand, cases involving small tumors (A through E) showed large variations in GTV dose coverage. PTV dose coverage for cases C through E and H through K from all planning strategies produced similar results (Table 3). Inclusion of treatment uncertainties reduced the differences of the PTV dose coverage from different planning strategies, for most cases. The most significant improvement in dose coverage is seen in small tumors when uncertainties are included; the smallest tumors (cases A and B) showing the maximum improvement in dose coverage (Table 2, Table 3); except with the AVG plan. AVG plans displayed poor PTV dose coverage for small tumors, especially cases A and B. In general, MIP3 and EE plans demonstrated better PTV dose coverage than CVCT and AVG plans. EE and AVG plans had the same density corrections for ITVs. However, EE plans yielded superior PTV dose coverage relative to AVG plans. The result that AVG plans need higher density corrections of ITVs agrees with the MD Anderson study [10]. The density corrections of ITVs in EE plans might introduce additional planning uncertainties. If a treatment planning system could capture ITVs from all breathing phases and superimpose them into EE images, EE planning can be a good planning strategy for proton lung treatment.

The mean lung dose from the plans with treatment uncertainties increases (Table 3). Treatment uncertainties have a more pronounced effect on mean lung dose for large tumors than for small tumors. Accordingly, larger ITV margins should be considered for very small tumors, such as for cases A and B. The CVCT plan for case G created the worst case scenario: lowest PTV dose coverage (PV_95_ =95.4%) and high lung dose (LV_20_ = 23.8 Gy, PM_LD_ = 10.8 Gy). The CVCT plan for case F, also showed poor PTV dose coverage. Both cases were characterized by large tumor movements with different motion patterns. The tumor in case F had the maximum movement along the superior-inferior direction, with the breathing period of 4 seconds. Case G had roughly the same movements along the superior–inferior and anterior–posterior directions, with the breathing period of 9 seconds. Motion artifacts in CVCT images might affect their ITV outlines, decrease the PTV dose coverage, and affect the ipsilateral mean lung dose differently.

Engelsman et al. [9] indicate that the ideal proton lung planning strategy using 4DCT scans is to design multiple plans using all phase images from a breathing cycle and combining them into one treatment plan. Apertures and compensators from the multiple plans have to be combined to create one aperture and compensator for each beam for treatment. The combined aperture will be similar to the aperture shaped to ITVs. The combined compensator will correspond to the maximum proton beam range required to cover the deepest target with the minimum range shift. The differences between the combined compensator and the compensator designed for ITVs in MIP3 plans may come from the variations in patient thickness along the proton beam path from different phases during a 4DCT scan. These variations from 4DCT scanning are minimal for a patient immobilized in a whole-body pod with free breathing. The treatment planning strategy of using multiple phase images or MIP3 images from 4DCT scans will be similar. However, the workload will increase significantly for designing multiple plans for all phase images compared to MIP3 plans from 4DCT scanning. MIP3 plans demonstrated the best PTV dose coverage and least sensitivity to tumor sizes and locations. Figure 4 illustrates this point for two very different cases: Case A (a-c) with smallest tumor size and case F (d-f) for largest tumor motion. This planning strategy is far superior than CVCT planning based on axial CT scans, predating the availability of 4DCT. The 4DCT scans are much faster (a minute or so) than axial scans (8–10 minutes). These scans capture motion more realistically, reduce motion artifacts, resulting in superior dosimetry, and considerably reduce patient exposure to CT radiation. MIP3 planning strategy investigated in this study is easy to implement in the clinic for routine proton lung treatment planning.

## Conclusions

Proton lung treatment can be improved by using 4DCT scanning to capture tumor motion correctly. In this study, three planning strategies, using MIP3, AVG, and EE images as the primary images from 4DCT scans, were demonstrably superior to CVCT planning from conventional axial CT scans. Among the twelve cases selected for the study, AVG plans displayed the lowest PTV dose coverage for small tumors and were most sensitive to tumor sizes and locations. CVCT planning demonstrated lower PTV dose coverage compared to MIP3, AVG, and EE plans for tumors with relatively large motion. EE plans were superior to CVCT and AVG plans. Inclusion of treatment uncertainties, in general, improved PTV dose coverage and reduced PTV dose differences for small tumors using different planning strategies. Among plans with and without treatment uncertainties, MIP3 plans demonstrated the best overall GTV and PTV dose coverage and least sensitivity to tumor sizes and locations. MIP3 planning based on 4DCT scans, investigated in this study, proved to be a superior planning strategy that could easily be implemented in the clinic for routine proton lung treatment planning.

## Competing interests

The authors declare that they have no competing interests.

## Authors’ contributions

NW designed the study, did all the analysis, and wrote the first draft of the manuscript. BP helped with revising and editing the manuscript and responding to the reviewers’ comments. AG helped with the initial design of the study, and provided useful comments for the final manuscript. DB provided the clinical insights about the study, and gave comments about the clinical usefulness of the study. All the authors read and approved the final manuscript.

## References

[B1] Bush DA Slater JD Bonnet R Cheek GA Dunbar RD Moyers M Slater JM Proton-beam radiotherapy for early-stage lung cancer Chest 1999 116 1313 1319 10.1378/chest.116.5.1313 10559093

[B2] Bush DA Proton Radiation Therapy for Lung Cancer: Is There Enough Evidence? Oncology 2010 24 11 21155458

[B3] Bush DA Slater JD Shin BB Cheek GA Miller DW Slater JM Hypofractionated proton beam radiotherapy for stage I lung cancer Chest 2004 126 1198 1203 10.1378/chest.126.4.1198 15486383

[B4] Moyers MF Miller DW Bush DA Slater JD Methodologies and tools for proton beam design for lung tumors Int J Radiat Oncol Biol Phys 2001 49 1429 1438 10.1016/S0360-3016(00)01555-8 11286851

[B5] Urie M Goitein M Wagner M Compensating for heterogeneities in proton radiation-therapy Phys Med Bio 1984 29 553 566 10.1088/0031-9155/29/5/008 6330772

[B6] Allien AM Siracuse KM Hayman JA Balter JM Evaluation of the influence of breathing on the movement and modeling of lung tumors Int J Radiat Oncol Biol Phys 2004 58 1251 1257 10.1016/j.ijrobp.2003.09.081 15001270

[B7] Pan T Lee TY Rietzel E Chen GTY 4D-CT imaging of a volume influence by the respiratory motion on multi-slice CT Med Phys 2004 31 333 340 10.1118/1.1639993 15000619

[B8] Pan T Comparison of helical and cine acquisitions for 4D-CT imaging with multislice CT Med Phys 2005 32 627 634 10.1118/1.1855013 15789609

[B9] Engelsman M Rietzel E Kooy HM Four-dimensional proton treatment planning for lung tumors Int J Radiat Oncol Biol Phys 2006 64 1589 1595 10.1016/j.ijrobp.2005.12.026 16580508

[B10] Kang Y Zhang X Chang JY Wang H Wei X Liao A Komaki R Cox JD Balter PA Liu H Zhu XR Mohan R Dong L 4D proton treatment planning strategy for mobile lung tumors Int J Radiat Oncol Biol Phys 2007 67 1589 1595 10.1016/j.ijrobp.2006.10.045 17293240

